# Whole‐Genome Characterization of Rodent Coronaviruses (RCoVs) in Thailand, 2024–2025

**DOI:** 10.1155/tbed/7053968

**Published:** 2026-08-12

**Authors:** Chanakarn Nasamran, Supassama Chaiyawong, Supanat Boonyapisitsopa, Napawan Bunpapong, Kamonpan Charoenkul, Alongkorn Amonsin

**Affiliations:** ^1^ Department of Veterinary Public Health, Faculty of Veterinary Science, Chulalongkorn University, Bangkok 10330, Thailand, chula.ac.th; ^2^ Emerging and Re-Emerging Infectious Diseases in Animals, Center of Excellence, Faculty of Veterinary Science, Chulalongkorn University, Bangkok 10330, Thailand, chula.ac.th; ^3^ Veterinary Diagnostic Laboratory, Faculty of Veterinary Science, Chulalongkorn University, Bangkok 10330, Thailand, chula.ac.th

**Keywords:** characterization, coronavirus, genetic, rodent, Thailand

## Abstract

Rodents are recognized as important reservoirs of coronaviruses, contributing to viral diversity and potential zoonotic emergence. However, data on rodent coronaviruses (RCoVs) in Thailand remained limited. This study aimed to determine the prevalence, genetic characteristics, and phylogenetic relationships of RCoVs circulating in rodent populations across Thailand. A total of 435 rodents were captured in 26 provinces across five geographic regions of Thailand between January 2024 and April 2025. Paired oral and rectal swabs (*n* = 870) from 435 rodents were collected and screened for RCoVs using one‐step RT‐PCR targeting the RNA‐dependent RNA polymerase (RdRp) gene. RCoVs were detected in 2.07% (9/435) of rodents or 1.15% (10/870) of swab samples, with higher detection in rectal swabs than oral swabs. All positive specimens were found exclusively in *Bandicota indica*. Of 10 positive swab samples, 5 were subjected to whole‐genome sequencing, and the rest to RdRp gene sequencing (*n* = 5). Phylogenetic and comparative genomic analyses were performed to determine genetic relationships and potential host adaptation. Our results showed that all Thai RCoVs belonged to the genus *Betacoronavirus*, subgenus *Embecovirus*, species *Betacoronavirus muris*, and exhibited conserved genomic organization and clustering with previously reported RCoVs from Asia. Thai RCoVs showed high nucleotide identity (up to ~97%) with RCoVs from China, Thailand, and Vietnam, indicating regional circulation and shared evolutionary origins. Analysis of the spike (S) protein revealed conserved receptor‐binding motifs associated with 9‐O‐acetylated sialic acid binding, alongside variability in key functional regions, including the S1/S2 cleavage site, suggesting adaptive evolution. In summary, this study demonstrated a low frequency of detection of genetically diverse RCoVs in three provinces of Thailand. RCoV genomic diversity and variability in S proteins warranted further investigation and underscored the importance of maintaining ongoing One Health surveillance to improve early detection and mitigate potential emerging coronavirus threats.

## 1. Introduction

Rodent coronavirus (RCoV) is an enveloped, positive‐sense, single‐stranded RNA virus within the family *Coronaviridae*, subfamily *Orthocoronavirinae*. RCoVs belong to the genera *Alphacoronavirus* and *Betacoronavirus*. Genus *Betacoronavirus* is the most common genus for RCoV, particularly the subgenus *Embecovirus*, including murine hepatitis virus (MHV), Longquan Aa mouse coronavirus (LAMV), as well as members of *Rattus coronavirus* from China (ChRCoV_HKU24), Myodes CoV 2JL14, and Longquan Rl rat coronavirus (LRLV) [1–4]. Genus *Alphacoronavirus* is often identified in wild rodents, including Lucheng Rn rat coronavirus (LRNV), AcCoV‐JC34 (found in *Apodemus chevrieri*), PLMg1, UKMa1, and UKRn1 (found in European bank voles/mice) [3, 4]. RCoVs have high genetic diversity, and rodents are considered important natural reservoirs for the evolution of coronaviruses through genetic recombination.

The genus *Betacoronavirus* is subdivided into four major subgenera: *Embecovirus*, *Sarbecovirus*, *Merbecovirus*, and *Nobecovirus*. The *Embecovirus* subgenus comprises RCoV, human coronavirus (HCoV, HCoV‐OC43, and HCoV‐HKU1), and bovine coronavirus (BCoV) [4, 5]. Notably, previous evolutionary studies suggested that HCoV‐OC43 and HCoV‐HKU1 may have originated from rodent‐borne ancestors, highlighting the zoonotic importance of rodent hosts [2, 6, 7]. The *Sarbecovirus* subgenus includes severe acute respiratory syndrome coronavirus (SARS‐CoV) and SARS‐CoV‐2 (the causative agent of COVID‐19) [8, 9]. Furthermore, *Merbecovirus* includes the Middle East respiratory syndrome coronavirus (MERS‐CoV) [10], whereas *Nobecovirus* primarily comprises bat‐associated viruses, such as Bat CoV HKU9 [11]. In 2025, the International Committee on Taxonomy of Viruses (ICTV) classified the subgenus *Embecovirus* into 5 species members, including species *Betacoronavirus gravedinis*, *Betacoronavirus ratti*, *Betacoronavirus muris*, *Betacoronavirus myodae*, and *Betacoronavirus hongkongense* (https://ictv.global/taxonomy).

The RCoV genome is ~27–32 kb. The genomic organization includes the structural proteins spike (S), envelope (E), membrane (M), and nucleocapsid (N), as well as several nonstructural proteins (NSPs). Many RCoVs in the genus *Embecovirus* have a unique hemagglutinin–esterase (HE) protein, which is not found in all coronavirus genera. Additionally, RCoV exhibits high mutation and recombination rates because rodents often carry multiple viral strains simultaneously, leading to the emergence of new recombinant lineages that may alter tissue tropism or host range [2, 6]. The zoonotic risk of RCoV is speculative. Historical and phylogenetic evidences suggest an evolutionary relationship between rodent and human CoVs. For example, a previous study reported that the HCoV strain HCoV‐OC43 likely originated from bovine CoV that may have originated in rodents. Furthermore, phylogenetic analysis showed that HCoV‐HKU1 was closely related to rodent *Embecovirus*, indicating that rodents were likely the original reservoir [7].

In Southeast Asia, particularly in Thailand, the risk of zoonotic spillover is highlighted by the frequent human–rodent interface associated with traditional dietary practices. Rodent consumption remained common in rural areas, especially in the northeastern and central regions, where field rats such as *Bandicota indica* and *R. argentiventer* are hunted as seasonal delicacies [12]. This practice involved intensive contact during trapping, manual butchering, and preparation, exposing hunters and handlers to rodent blood and respiratory secretions and excreta [13]. Such activities provided a direct pathway for the transmission of rodent‐borne pathogens, including coronaviruses, to human populations. As of May 2026, 194 whole‐genome sequences of RCoV have been deposited in the GenBank database. However, whole‐genome sequences of RCoVs in Thailand are not available in the database. This study aimed to determine the prevalence, genetic characteristics, and phylogenetic relationships of RCoVs circulating in rodent populations across Thailand. This study is the first to report whole‐genome sequences of Thai RCoVs recovered from rodents in Thailand.

## 2. Material and Methods

### 2.1. Ethical Approval

This study was conducted with approval from the Chulalongkorn University Animal Care and Use Committee (IACUC), under Protocol CUIACUC #2431085. All procedures were conducted in accordance with institutional and ARRIVE guidelines. Oral and rectal swab samples were collected from animal carcasses by licensed veterinarians or trained staff. Personal protective equipment was used during the sample collection. Data collected during the study were anonymized to protect the identities of farmers or rodent sellers.

### 2.2. Study Sites and Sample Collection

**Figure 1:**
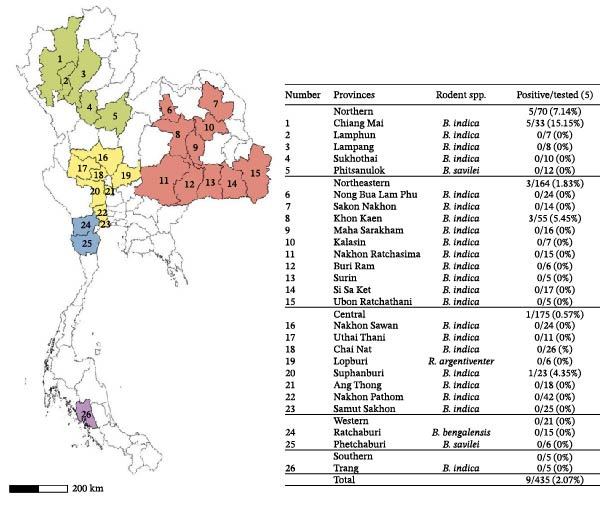
Sample collection from rodents and the prevalence of RCoV in each province, Thailand.

In this study, a cross‐sectional survey of RCoV in rodents in Thailand was conducted from January 2024 to April 2025. In total, 435 rodents were captured from rural areas across 26 provinces in Thailand. The 26 provinces are located across all regions of Thailand, including northern, northeastern, central, western, and southern provinces (northern provinces: Chiang Mai, Lamphun, Lampang, Sukhothai, and Phitsanulok; northeastern provinces: Nong Bua Lam Phu, Sakon Nakhon, Khon Kaen, Maha Sarakham, Kalasin, Nakhon Ratchasima, Buri Ram, Surin Si Sa Ket, and Ubon Ratchathani; central provinces: Nakhon Sawan, Uthai Thani, Chai Nat, Lopburi, Suphanburi, Ang Thong, Nakhon Pathom, and Samut Sakhon; western provinces: Ratchaburi and Phetchaburi; and southern province: Trang) (Figure 1). The animals were trapped or captured in rice fields in rural areas or near villages by farmers or sellers. Animal carcasses were transported to the Center of Excellence for Emerging and Re‐emerging Infectious Diseases in Animals (CUEIDAs) at Chulalongkorn University at temperatures ranging from 4 to −15°C, depending on the distance traveled. The selection criteria for rodent carcasses for sample collection include random sampling of healthy, freshly collected carcasses. At the laboratory, paired oral swabs (*n* = 435) and rectal swabs (*n* = 435) were collected from each of the 435 rodents and placed in 1 mL of phosphate‐buffered saline (PBS). All swab samples were stored at −20°C for virus detection until further use.

### 2.3. Rodent Species Identification

Rodent species identification was based on morphological characteristics, including body weight, body length, tail length, and skull length. To confirm these morphological assignments, molecular species identification was performed on 26 representative rodent samples selected from different geographic locations. Conventional PCR using primers specific to the mitochondrial cytochrome *b* (*cytb*) gene was used to amplify genomic DNA from the rodent samples [14, 15]. The 457 bp amplified product was submitted for Sanger sequencing. The nucleotide sequences were analyzed using the basic local alignment search tool (BLAST) against the NCBI database to verify rodent species identity with high taxonomic confidence.

### 2.4. Detection of RCoVs

To extract viral RNA, all oral swabs (*n* = 435) and rectal swabs (*n* = 435) were subjected to RNA extraction using the GeneAll GENTi Viral DNA/RNA Extraction Kit (GeneAll; Lisbon, Portugal) on a GENTi 32 automated extractor. 200 μL of each swab sample was added to the extraction tube, and RNA extraction was carried out following the manufacturer’s instructions. Extracted RNA samples were stored at −80°C until further analysis. The RNA samples were tested for coronavirus using a one‐step RT‐PCR specific to the RNA‐dependent RNA polymerase (RdRp) gene [16]. The primer sequences used in this study were forward primer: 5^′^‐GGNTGGGAYTAYCCNAARTGYGA‐3^′^ and reverse primer: 5^′^‐CCACACCATCATCAGATAGAATCA‐3^′^). In brief, the PCR was performed using the SuperScript III One‐Step RT‐PCR System with Platinum Taq DNA Polymerase (Invitrogen; California, USA). In a 50 μL PCR reaction, 5 μL of RNA, 0.4 μM of each primer, 1× Reaction Mix, 4% v/v or 2 μL of SuperScript III RT/Platinum Taq Mix, and nuclease‐free water to a total volume of 50 μL were used. The PCR conditions included an initial step at 50°C for 30 min and then 94°C for 2 min. It was followed by 40 cycles of 94°C for 15 s, 45°C for 30 s, and 68°C for 30 s. A final extension was performed at 68°C for 7 min. PCR products were analyzed by 1.5% agarose gel electrophoresis, with an expected product size of 445 bp.

### 2.5. RdRp Gene and Whole‐Genome Sequencing of RCoV

All positive RCoV swab samples (*n* = 10) were subjected to RdRp gene sequencing using Sanger sequencing. To perform sequencing, the PCR products were purified using the NucleoSpin Gel and PCR Clean‐up kit (Macherey‐Nagel, Düren, Germany) and submitted to BIONIC Co., Ltd. for sequencing. The nucleotide sequences were validated, and consensus sequences were generated in the FASTA format using the SeqMan program (DNASTAR, Madison, WI, USA).

Five out of 10 RCoVs were selected for whole‐genome sequencing. The swab samples were selected based on RNA quality, sampling location, collection date, and sample type. Whole‐genome sequencing was performed using Oxford Nanopore Technologies (ONT, Oxford, UK). The cDNA was synthesized using the SuperScript IV First‐Strand Synthesis System (Invitrogen) with random hexamers, oligo(dT), and specific RCoV primers. The DNA fragments were amplified by nested PCR using Platinum *Taq* DNA Polymerase, High Fidelity (Invitrogen, CA, USA) for the first round of PCR and HotStarTaq Master Mix (Qiagen; Hilden, Germany) for the second round of PCR. Sequencing primers used in the study were designed from reference CoV sequences with Accession Numbers KY370049, MT085168, and MT085171 (Table S1).

To perform Oxford Nanopore sequencing, pooled PCR products were purified using NucleoSpin Gel and PCR Clean‐up (Machery‐Nagel, Germany) and quantified using a NanoDrop (Thermo Fisher Scientific, Waltham, MA, USA). DNA libraries were prepared using the Native Barcoding Kit 24 V14 (SQK‐NBD114.24). The DNA libraries were run on an R10.4.1 MinION flow cell. Basecalling was performed using Guppy (v6.5.7) with a minimum read quality filter Q‐score > 7 and converted Fast5 to Fastq format. Reads were aligned to reference RCoV sequences (MHV [NC 001846], Parker’s rat CoV [FJ938068]) using Minimap2. The coverage was determined via Qualimap. The draft genome was polished using Racon and refined with Medaka (v2.0.0). The final whole‐genome consensus sequences were generated using samtools (v1.21) from the polished alignments. Positions with a depth coverage below 20× or ambiguous alignments were marked as “N.” Subsequently, these low‐coverage regions or gaps, along with critical S gene residues such as the S1/S2 cleavage site, were targeted and completely resolved via Sanger sequencing to achieve full consensus accuracy. Final whole‐genome sequences were provided in the FASTA format. The whole‐genome sequences of Thai RCoV (*n* = 5) were analyzed for open reading frames (ORFs) using the NCBI ORF Finder (https://www.ncbi.nlm.nih.gov/orffinder/) with the standard genetic code to determine gene positions.

### 2.6. Phylogenetic Analysis and Genetic Analysis of the RdRp Gene and Whole‐Genome Sequences of RCoV

Phylogenetic analysis was performed by comparing the RdRp, S, E, M, and N genes of the Thai RCoV with those of reference coronaviruses. The reference coronaviruses used in phylogenetic analysis include representative strains of *Alphacoronavirus* (*n* = 16;, e.g., FCoV, PEDV, TGEV, HCoV‐NL63, BtCoV‐512, and BtCoV‐HKU8) and *Betacoronavirus* (*n* = 51; e.g., MHV, Parker’ rat CoV, SDAV. BCoV, HCoV‐OC43, HCoV‐HKU1, HCoV‐4408, RCoV‐HKU24, SARs‐CoV, SARs‐CoV‐2, MERs‐CoV, BtCoV‐HKU9, and available RCoVs), obtained from the GenBank database and representing different host species. Multiple sequence alignments of RdRp, S, E, M, and N genes were performed using ClustalW within MEGA V.11.0. Phylogenetic trees were generated in MEGA v.11.0 with 1,000 bootstrap replicates using the neighbor‐joining method and the maximum composite likelihood model. A gamma distribution (+G) was employed, and all positions containing gaps were addressed using pairwise deletion.

Pairwise comparisons for whole‐genome sequences and each gene (RdRP, ORF1ab, S, E, M, and N) of RCoV were performed in MEGA V.11.0 with those of the reference coronaviruses. The genetic analysis focused only on comparing the amino acid sequences of the S protein. To analyze, the S protein of Thai RCoVs was aligned with that of the reference *Betacoronavirus* using the ClustalW algorithm in MEGA V.11.0. Conserved receptor‐binding sites (RBS) for CARCAM1 and 9‐O‐acetyl sialic acid were analyzed to determine potential host receptor binding. In brief, for CARCAM1 and 9‐O‐acetyl sialic acid, the Thai RCoVs were compared with HCoV‐HKU1 (NC 006577), HCoV‐OC43 (AY391777), BCoV‐DB2 (DQ811784), CoV‐HKU24 (NC 026011), and MHV‐A59 (NC 001846). In this study, the whole‐genome organization of Thai RCoVs (*n* = 5) was constructed and compared with that of representative viruses from *Alphacoronavirus* and *Betacoronavirus* (*embecovirus*, *sarbecovirus*, *merbecovirus*, and *nobecovirus*). The genomic organization was generated using the gggenes package (https://wilkox.org/gggenes/) within RStudio (V.20.26.04.0 + 256, Posit Software, PBC, Boston, USA). Precise gene positions, lengths, and orientations of the references were extracted from GenBank‐annotated files to ensure an accurate comparison.

## 3. Recombination Analysis

Recombinant Detection Program (RDP5) and Simplot v.3.5.1 were used to detect potential recombination events for Thai RCoVs in this study. In brief, initial screening for recombination, identification of potential parental sequences, and localization of recombination breakpoints were performed using RDP5. Six distinct algorithms implemented in RDP5 (including RDP, GENECONV, Bootscan, MaxChi, Chimera, and SiScan) were executed with default parameters. Recombination events were considered acceptable when statistically supported by at least four of these six methods, with a *p*‐value threshold of <0.05 [17]. To visually confirm the identified recombination events, Bootscan analyses were conducted using SimPlot v.3.5.1. The Bootscan analysis was performed with a sliding window size of 500 bp and a step size of 50 bp under the Kimura (two‐parameter) distance model [18].

## 4. Statistical Analysis

Statistical analyses were conducted using SPSS Version 30.0.0.0 (IBM Corp., Armonk, NY, USA). Descriptive statistics were summarized as counts and percentages. Fisher’s exact test was used to evaluate the association between RCoV prevalence and categorical variables (rodent species, sex, location, and swab sample type). For data precision, 95% confidence intervals (CI) were calculated for all reported proportions. A *p*‐value of 0.05 was considered significant.

## 5. Result

### 5.1. Sample Collection

A total of 435 rodents were collected from 26 provinces in the northern, northeastern, central, western, and southern regions of Thailand. From each animal, paired oral (*n* = 435) and rectal (*n* = 435) swabs were obtained, resulting in 870 total swab samples. Most rodent specimens were collected from rural agricultural settings, particularly rice field environments. Rodent specimens were geographically distributed, with the highest numbers collected from the central (*n* = 175) and northeastern (*n* = 164) regions, followed by the northern (*n* = 70), western (*n* = 21), and southern (*n* = 5) regions. Rodent species identification indicated that the predominant species sampled was *B. indica*, with smaller numbers of *B. savilei*, *B. bengalensis*, and *R. argentiventer* (Table 1 and Figure 1).

**Table 1:** Sample collection from rodents and the RCoV positivity by provinces in Thailand.

No.	Location	Region	Number of animals	Number of swab sample	RCoV detection (positive/tested [%])			
					Rectal swab	Oral swab	Positive swab sample	Positive animal
1	Chiang Mai	Northern	33	66	4/33 (12.12%)	1/33 (3.03%)	5/66 (7.58%)	5/33 (15.15%)
2	Lamphun	Northern	7	14	0/7	0/7	0/14	—
3	Lampang	Northern	8	16	0/8	0/8	0/16	—
4	Sukhothai	Northern	10	20	0/10	0/10	0/20	—
5	Phitsanulok	Northern	12	24	0/12	0/12	0/24	—
6	Nong Bua Lam Phu	Northeastern	24	48	0/24	0/24	0/48	—
7	Sakon Nakhon	Northeastern	14	28	0/14	0/14	0/28	—
8	Khon Kaen	Northeastern	55	110	3/55 (5.45%)	1/55 (1.82%)	4/110 (3.64%)	3/55 (5.45%)^a^
9	Maha Sarakham	Northeastern	16	32	0/16	0/16	0/32	—
10	Kalasin	Northeastern	7	14	0/7	0/7	0/14	—
11	Nakhon Ratchasima	Northeastern	15	30	0/15	0/15	0/30	—
12	Buri Ram	Northeastern	6	12	0/6	0/6	0/12	—
13	Surin	Northeastern	5	10	0/5	0/5	0/10	—
14	Si Sa Ket	Northeastern	17	34	0/17	0/17	0/34	—
15	Ubon Ratchathani	Northeastern	5	10	0/5	0/5	0/10	—
16	Nakhon Sawan	Central	24	48	0/24	0/24	0/48	—
17	Uthai Thani	Central	11	22	0/11	0/11	0/22	—
18	Chai Nat	Central	26	52	0/26	0/26	0/52	—
19	Lopburi	Central	6	12	0/6	0/6	0/12	—
20	Suphanburi	Central	23	46	1/23 (4.35%)	0/23	1/46 (2.17%)	1/23 (4.35%)
21	Ang Thong	Central	18	36	0/18	0/18	0/36	—
22	Nakhon Pathom	Central	42	84	0/42	0/42	0/84	—
23	Samut Sakhon	Central	25	50	0/25	0/25	0/50	—
24	Ratchaburi	Western	15	30	0/15	0/15	0/30	—
25	Phetchaburi	Western	6	12	0/6	0/6	0/12	—
26	Trang	Southern	5	10	0/5	0/5	0/10	—
	Total	—	435	870	8/435 (1.84%)	2/435 (0.46%)	10/870 (1.15%)^a^	9/435 (2.07%)

^a^One animal was positive for both oral and rectal swabs.

### 5.2. Identification of RCoV

All swab samples were tested for RCoVs using a one‐step RT‐PCR assay targeting the RdRp gene. Out of 435 rodents tested, 9 animals (9/435; 2.07%) were positive for RCoV. By sample, of 870 swab samples tested, 10 swab samples (10/870; 1.15%) were positive for RCoV. The swab samples were more positive in rectal swabs (8/435; 1.84%, 95% CI: 0.93−3.59) than in oral swabs (2/435; 0.46%, CI: 0.13−1.66). However, this difference was not statistically significant (Fisher’s test, *p* = 0.107). Note that all 10 positive swab samples were found only in *B. indica*. By geographic location, RCoVs were identified in *B. indica* populations, mainly in the northern, northeastern, and central regions of the country. The highest prevalence was observed in the northern region, Chiang Mai (*n* = 5/70; 7.14%), followed by the northeastern region, Khon Kaen (*n* = 3/164; 1.83%), and the central region, Suphanburi (*n* = 1/175; 0.57%). No positive animals were detected in the western or southern regions. Statistical analysis showed a significant association between RCoV positivity and geographic location by region (*p* = 0.026). However, the observed regional differences should be interpreted cautiously due to uneven sampling and the low number of positives. Notably, no significant associations were observed in sex (*p* = 0.505) or rodent species (*p* = 1.000) (Tables 2 and 3).

**Table 2:** Analysis of the associated risk factors of RCoV positivity among rodent populations (*N* = 435).

Factor		RCoV status		Total	Prevalence	95% CI	95% CI	Fisher‘s
		Positive animals	Negative animals		(%)	Lower	Upper	*p*‐Value^a^
Sex	M	3	208	—	1.42	0.48	4.10	—
	F	6	218	435	2.68	1.23	5.72	0.505
Location	Northern region	5	65	—	7.14	3.09	15.66	—
	Northeastern region	3	161	—	1.83	0.62	5.24	—
	Central region	1	174	—	0.57	0.10	3.17	—
	Other	0	26	435	0.00	0.00	12.87	0.026 ^∗^
Rodent species	*B. indica*	9	397	—	2.22	1.17	4.16	—
	*B. savilei*	0	18	—	0.00	0.00	17.59	—
	*B. bengalensis*	0	5	—	0.00	0.00	43.45	—
	*R. argentiventer*	0	6	435	0.00	0.00	30.03	1.000
Overall	—	9	426	435	2.07	—	—	—

^a^Statistical analysis was performed using Fisher’s exact test.

^∗^Statistical significance at *p*  < 0.05.

**Table 3:** Analysis of the association between RCoV prevalence and swab sample types.

Factor		RCoV status		Total	Prevalence	95% CI	95% CI	Fisher ‘s
		Positive swabs	Negative swabs		(%)	Lower	Upper	*p*‐Value
Sample type	Rectal swab	8	427	435	1.84	0.93	3.59	—
	Oral swab	2	433	435	0.46	0.13	1.66	0.107
	Overall	10	860	870	1.15	—	—	—

*Note:* Statistical analysis was performed using Fisher’s exact test.

### 5.3. Characterization of RCoV

**Figure 2:**
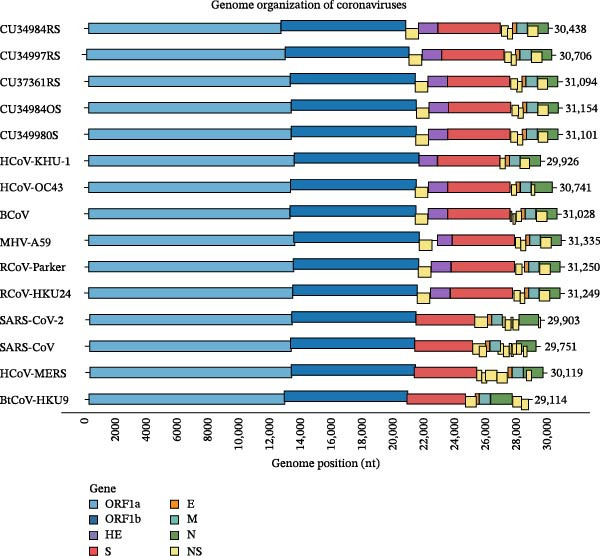
Genome structure of Thai RCoVs compared to the Betacoronaviruses.

Five positive swab samples were selected for whole‐genome sequencing based on RNA quality, geographic distribution, and sampling time. The sequenced viruses originated from multiple regions, including Chiang Mai (northern), Khon Kaen (northeastern), and Suphanburi (central). In addition, five swab samples underwent sequencing of the RdRp gene (Table 4). In this study, we successfully obtained complete whole‐genome sequences for five Thai RCoVs and partial RdRp gene sequences for the remaining five Thai RCoVs. Details of whole‐genome sequencing using Oxford Nanopore sequencing, mapping statistics, and coverage depth for Thai RCoVs are provided (Table S2 and Figure S1). The nucleotide sequences of the Thai RCoVs were deposited in the GenBank database under Accession Numbers PZ438032–PZ438041. The genomic organization of Thai RCoVs (CU34984RS, CU34984OS, CU34997RS, CU34998OS, and CU37361RS) was compared with representative members of the *Alphacoronavirus* and *Betacoronavirus* genera. All Thai RCoVs exhibited a conserved gene order typical of the *Betacoronavirus* genus (5^′^‐ORF1ab‐HE‐S‐E‐M‐N‐3^′^). The arrangement of the accessory genes located in two regions, between the ORF1ab and S proteins and between the S and E proteins, as well as those downstream of the N protein, showed high similarity with members of the *Embecovirus* subgenus, specifically HCoV‐HKU1, HCoV‐OC43, and BCoV (Figure 2).

**Table 4:** Detailed description of the Thai RCoV characterized by whole‐genome and RdRp sequencing in this study.

Sample ID	Location	Region	Date	Swab sample type	Genotype	Sequencing	GenBank number
CU34984RS^a^	Khon Kaen	Northeastern	January 24	Rectal swab	Beta A	WGS	PZ438032
CU34984OS^a^	Khon Kaen	Northeastern	January 24	Oral swab	Beta A	WGS	PZ438033
CU34997RS	Chiang Mai	Northern	January 24	Rectal swab	Beta A	WGS	PZ438034
CU34998OS	Chiang Mai	Northern	January 24	Oral swab	Beta A	WGS	PZ438035
CU36371RS	Suphanburi	Central	January 25	Rectal swab	Beta A	WGS	PZ438036
CU34987RS	Khon Kaen	Northeastern	January 24	Rectal swab	Beta A	RdRp	PZ438037
CU34988RS	Khon Kaen	Northeastern	January 24	Rectal swab	Beta A	RdRp	PZ438038
CU34999RS	Chiang Mai	Northern	January 24	Rectal swab	Beta A	RdRp	PZ438039
CU35000RS	Chiang Mai	Northern	January 24	Rectal swab	Beta A	RdRp	PZ438040
CU35002RS	Chiang Mai	Northern	January 24	Rectal swab	Beta A	RdRp	PZ438041

^a^One animal was positive for both oral and rectal swabs.

Based on nucleotide identity comparisons, all Thai RCoVs were identified as *Betacoronavirus* subgenus *Embecovirus*, species *Betacoronavirus muris* (lineage A), which has a unique genomic structure characterized by the presence of the hemagglutinin–esterase (HE) gene. Whole‐genome comparison demonstrated that the Thai RCoV‐CU34984RS shared the highest nucleotide identity with other rat coronaviruses (RtCoVs) belonging to the *Embecovirus* subgenus. For example, Thai RCoV‐CU34984RS exhibited 89.24%–92.07% whole‐genome identity among Thai RCoVs. Thai RCoV‐CU34984RS also showed similarly high identities with RtRt‐CoV‐Tk2011 from China (92.66%), RtRe‐CoV‐Tl2006A from Thailand (92.76%), and MuCoV‐VZ‐16715 from Vietnam (89.64%). In contrast, substantially lower whole‐genome nucleotide identities were observed with other embecoviruses from humans, cattle, pigs, and mice (71.35%–78.02%), as well as with sarbecoviruses, merbecoviruses, nobecoviruses, and Alphacoronaviruses (45.96%–53.40%). Comparative genomic analysis of S, E, M, N, and HE genes also showed high nucleotide identities among Thai RCoVs, ranging from 80.05% to 97.04 %. Thai RCoVs shared moderate‐to‐high nucleotide identity with previously reported viruses in the *Embecovirus* subgenus. The highest similarity was observed with RCoVs from Asia, including RtCoVs from China (RtRt‐CoV‐Tk2011), with up to 97.19% nucleotide identity in structural genes; RtCoV from Thailand (RtRe‐CoV‐Tl2006A), with up to 95.09% nucleotide identity; and RtCoV from Vietnam (MuCoV‐VZ‐16715), with up to 95.41% nucleotide identity. In contrast, the Thai RdCoVs shared only 68.55%–74.10% identity of the S gene with *Embecovirus* references (such as HCoV‐HKU1, HCoV‐OC43, and MHV) and showed low identity (<48%) to *Sarbecovirus* (lineage B), *Merbecovirus* (lineage C), and *Nobecovirus* (lineage D) (Table 5).

**Table 5:** Nucleotide identities of whole‐genome sequences of Thai RCoV (CU34984RS) compared with references Alphacoronavirus and Betacoronavirus.

Virus ID	Host	Location	Year	Group	% nucleotide identity						
					WG	S gene	E gene	M gene	N gene	HE gene	RdRp gene
This study											
CU34984RS	Rat	Thailand	2024		100.00	100.00	100.00	100.00	100.00	100.00	100.00
CU34984OS	Rat	Thailand	2024		92.07	80.18	96.86	96.10	97.04	95.73	94.77
CU34997RS	Rat	Thailand	2024		89.24	90.00	94.51	93.36	92.76	86.55	94.70
CU34998OS	Rat	Thailand	2024		89.35	90.07	94.90	93.36	92.54	86.55	94.52
CU36371RS	Rat	Thailand	2025		90.73	80.05	96.86	96.25	94.83	96.05	95.71
Reference											
HCoV‐HKU1 (NC 006577)	Human	China	2003	Embecovirus	77.93	74.10	69.88	78.13	74.83	67.10	86.81
HCoV‐4408 (FJ415324)	Human	Germany	1988	Embecovirus	75.12	73.00	75.90	81.01	73.35	70.53	87.14
BCoV‐DB2 (DQ811784)	Bovine	USA	1983	Embecovirus	75.12	72.92	75.50	81.01	73.57	70.61	87.03
PHEV‐VW572 (DQ011855)	Pig	Belgium	2005	Embecovirus	75.06	71.77	75.90	80.87	72.36	71.01	87.06
HCoV‐OC43‐ATCC VR‐759 (AY391777)	Human	USA	1967	Embecovirus	75.19	72.75	75.50	81.59	73.05	69.32	87.03
CoV‐HKU24‐ R05005I (NC 026011)	Rat	China	2012	Embecovirus	71.35	68.55	77.11	82.97	70.91	68.52	84.40
MHV‐A59 (NC 001846)	Mouse	USA	1961	Embecovirus	78.02	72.62	80.95	81.51	79.75	64.73	88.86
RtRe‐CoV‐Tl2006A (MT085171)	Rat	Thailand	2006	Embecovirus	92.76	80.75	91.37	93.94	94.90	95.09	94.77
RtRt‐CoV‐Tk2011 (MT085168)	Rat	China	2011	Embecovirus	92.66	80.18	96.08	95.82	97.19	94.36	94.83
MuCoV‐VZ BetaCoV 16715 52 (MH687968)	Rat	Vietnam	2014	Embecovirus	89.64	88.91	90.59	90.76	90.81	95.41	93.23
SARS‐CoV‐civet007 (/AY572034)	Civet	China	2004	Sarbecovirus	50.47	47.16	43.72	50.45	45.04	n/a	64.25
SARS‐CoV‐2‐Wuhan‐Hu‐1 (MN908947)	Human	China	2019	Sarbecovirus	50.81	47.16	43.42	49.33	45.21	n/a	65.33
SARS‐CoV‐Tor2 (NC 004718)	Human	China	2003	Sarbecovirus	50.46	47.02	43.72	50.45	44.96	n/a	64.25
MERS‐CoV‐Munich (KF192507)	Human	Arab Emirates	2013	Merbecovirus	53.40	48.41	46.99	54.39	47.08	n/a	65.91
MERS‐CoV‐Al‐Hasa1 (KF186567)	Human	Saudi Arabia	2013	Merbecovirus	53.39	48.47	46.99	54.39	47.08	n/a	65.98
CoV‐GCCDC1 356 (NC 030886)	Rat	China	2014	Nobecovirus	48.50	46.43	40.35	47.60	45.93	n/a	64.22
BtCoV‐HKU9‐1 (EF065513)	Bat	China	2005	Nobecovirus	50.57	47.19	44.74	49.63	45.48	n/a	66.05
FIPV‐WSU‐79/1146 (AY994055)	Cat	USA	—	Alphacoronavirus	45.96	42.51	40.17	46.09	42.12	n/a	60.53
BtCoV‐512 (DQ648858)	Bat	China	2005	Alphacoronavirus	47.34	41.62	44.00	50.45	39.77	n/a	60.57
PEDV‐CV777 (NC 003436)	Pig	Belgium	1977	Alphacoronavirus	46.56	41.59	40.00	49.03	39.68	n/a	60.49

### 5.4. Phylogenetic Analysis of RCoV

**Figure 3:**
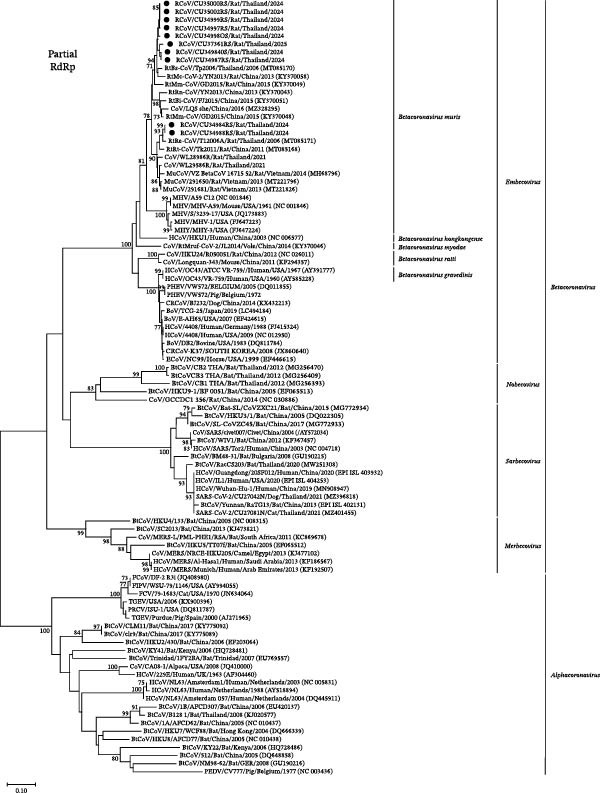
Phylogenetic tree of the partial RdRp gene. The phylogenetic tree was constructed using the neighbor‐joining algorithm and the maximum composite likelihood model, with 1000 bootstrap replicates.

Phylogenetic analysis of partial RdRp gene sequences showed that all Thai RCoVs clustered within the *Betacoronavirus* genus, *Embecovirus* subgenus, and *Betacoronavirus muris* species (lineage A). Thai RCoVs were grouped into two phylogenetic subclusters within the Embecovirus lineage. Specifically, eight Thai RCoVs (*n* = 8) (CU34984OS, CU34987RS, CU34997RS, CU34998OS, CU34999RS, CU35000RS, CU35002RS, and CU36371RS) cluster closely with rodent‐associated coronaviruses previously reported in Asia, YN2013 from China (KY370058) and Tp2006 from Thailand (MT085170). In contrast, the remaining two Thai RCoV strains (CU34984RS and CU34988RS) show a closer phylogenetic relationship to Tk2011 from China (MT085168) and Tl2006A from Thailand (MT085171) (Figure 3).

**Figure 4:**
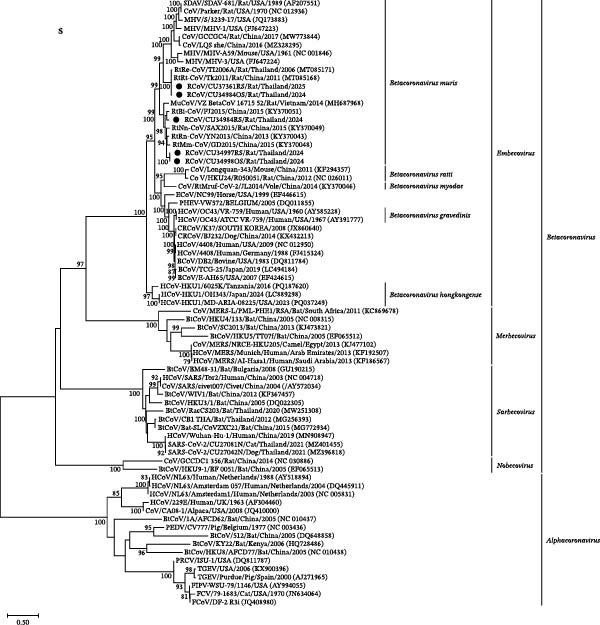
Phylogenetic tree of the S gene. The phylogenetic tree was constructed using the neighbor‐joining algorithm and the maximum composite likelihood model, with 1000 bootstrap replicates.

For the S, E, M, and N genes, the Thai RCoVs consistently clustered within the *Betacoronavirus muris* species, *Embecovirus* subgenus of the genus *Betacoronavirus*. Bootstrap replicates provided strong support for clustering Thai RCoVs with known *Betacoronavirus muris*, including MHV and RtCoVs. No clustering with *Alphacoronavirus* or other *Betacoronavirus* subgenera (e.g., *Sarbecovirus* or *Merbecovirus*) was observed, confirming the taxonomic placement of the detected viruses. In the S gene tree, the Thai RCoVs divide into two subclades: one group (CU34984RS, CU34997RS, and CU34998OS) clusters closely with the Chinese RCoVs—YN2013 (KY370043) and GD2015 (KY370048), while another group (CU37361RS and CU34984OS) was clustered with Chinese RCoV—Tk2011 (MT085168) and Thai RCoV—TI2006A (MT085171) (Figure 4). In contrast, the M, N, and E gene phylogenies showed a different arrangement, in which the Thai RCoVs CU37361RS, CU34984RS, and CU34984OS cluster together near the reference Thai RCoV (Tp2006) and Chinese RCoV (Tk2011). Meanwhile, CU34997RS and CU34998OS formed a separate cluster more closely related to the Chinese strain (SAX2015) (Figure S2).

### 5.5. Genetic Analysis of RCoV

Analysis of the S protein revealed conserved and variable residues across receptor‐binding regions. The results showed that most Thai RCoVs retained conserved motifs in the N‐terminal domain (NTD) of 9‐O‐acetylated sialic acid, similar to those of HCoV‐HKU1 and BCoV, indicating potential conservation of receptor usage. Thai RCoVs contained W90 (tryptophan) and L80 (leucine), K81 (lysine), and T83 (threonine) amino acid residues for 9‐O‐acetylated sialic acid, which were similar to HCoV‐OC43, BCoV, and HCoV‐HKU1 strains in humans and cattle [19, 20]. At the CEACAM1 receptor‐binding site, Thai RCoVs showed partial conservation with other *Embecoviruses* but exhibited distinct amino acid substitutions compared to those of HCoV and BCoV. Differences at residues 89, 160, 172, and 174 relative to those of reference human, bovine, and rat RCoVs indicated that Thai RCoVs showed divergence at the CEACAM1‐binding site [21]. For the S1/S2 cleavage site (furin cleavage site), variability was observed at positions 806−810 (RRKRR or RRARR). Polybasic residues are essential for S protein priming and M fusion [22–24] (Table 6). Overall, the genetic and amino acid analyses confirmed that Thai RCoVs are closely related to rodent‐associated *Embecoviruses* and exhibit host‐specific adaptations distinct from those of human‐infecting coronaviruses. The observed S features warrant further functional investigation and continued RCoV surveillance.

**Table 6:** Genetic analysis of RCoV at the receptor‐binding site (CEACAM1, 9‐O‐acetyl sialic acid), S1/S2 cleavage site.

Virus ID	Host	Location	Year	Amino acid position^a^													
				Receptor binding site of CEACAM1													
				15	19	20	22		23	24	25	27	29	89	160	172	174
CU34984RS	Rat	Thailand	2024	V	L	K	T	—	T	P	S	I	D	T	Y	Q	Q
CU34984OS	Rat	Thailand	2024	V	F	K	T	—	T	L	N	I	D	T	Y	Q	Q
CU34997RS	Rat	Thailand	2024	A	L	K	T	—	T	S	S	I	D	T	Y	Q	Q
CU34998OS	Rat	Thailand	2024	A	L	K	T	—	T	S	S	I	D	T	Y	Q	Q
CU36371RS	Rat	Thailand	2025	V	F	K	T	—	T	L	N	I	D	T	Y	Q	Q
HCoV‐HKU1 (NC 006577)	Human	China	2003	V	F	N	T	—	N	F	A	I	D	T	Y	S	N
BCoV‐DB2 (DQ811784)	Bovine	USA	1983	V	L	K	T	—	—	T	V	I	D	T	Y	Q	V
HCoV‐OC43‐ATCC VR‐759 (AY391777)	Human	USA	1967	V	L	K	T	S	D	T	S	I	D	R	Y	H	K
CoV‐HKU24‐ R05005I (NC 026011)	Rat	China	2012	I	F	K	T	—	—	Q	S	I	S	T	Y	G	P
MHV‐A59 (NC 001846)	Mouse	USA	1961	Y	F	R	I	—	Q	L	V	S	G	L	L	N	L

Virus ID	Host	Location	Year	Amino acid position													
				9‐O‐acetyl sialic acid receptor‐binding site							S1/S2 cleavage site						
				27	31	80	81	83	86	90		806		807	808	809	810
CU34984RS	Rat	Thailand	2024	N	T	L	K	T	L	W		R		R	K	R	R
CU34984OS	Rat	Thailand	2024	N	T	F	K	T	L	W		R		R	A	R	R
CU34997RS	Rat	Thailand	2024	N	T	L	K	T	L	W		R		R	K	R	R
CU34998OS	Rat	Thailand	2024	N	T	L	K	T	L	W		R		R	K	R	R
CU36371RS	Rat	Thailand	2025	N	T	L	K	T	L	W		R		R	A	R	R
HCoV‐HKU1 (NC006577)	Human	China	2003	N	T	L	K	T	L	W		R		R	K	R	R
BCoV‐DB2 (DQ811784)	Bovine	USA	1983	N	T	L	K	T	L	W		R		R	S	R	R
HCoV‐OC43‐ATCC VR‐759 (AY391777)	Human	USA	1967	N	T	L	K	S	L	W		R		R	S	R	G
CoV‐HKU24‐ R05005I (NC026011)	Rat	China	2012	N	S	L	K	T	L	W		W		R	R	K	R
MHV‐A59 (NC001846)	Mouse	USA	1961	N	V	L	T	T	V	W		R		R	R	D	R

^a^Numbering based on MHV‐A59.

In this study, we performed screening for potential recombination events using RDP5. Five whole‐genome sequences were analyzed using RDP5 and visually conformed by the Bootscan analysis of Simplot v.3.5.1. Our result showed that a recombination event was identified within the S and NS4 genes of Thai RCoV‐CU34984RS; the recombination event was supported by RDP5 detection algorithms with a significant *p*‐value: RDP (2.475 × 10^−109^), GENECONV (1.380 × 10^−175^), Bootscan (1.380 × 10^−175^), MaxChi (1.127 × 10^−44^), Chimaera (4.310 × 10^−46^), and SiScan (4.310 × 10^−46^). Recombinant analysis identified the Chinese RCoV strains RtRt‐CoV/Tk2011 and RtBi‐CoV/FJ2015 as the major and minor parents, respectively. Simplot analysis showed that the majority of the Thai RCoV‐CU34984RS genome is derived from the RCoV‐Tk2011 backbone (major parent), except for the nucleotide positions at 24470‐27327, which were acquired from the Chinese RCoV‐FJ2013 (minor parent). Phylogenetic analysis of the region within and without this recombination region also supported this recombination pattern (Figure S3).

## 6. Discussion

### 6.1. Epidemiology and Prevalence of RCoVs

This study demonstrated a relatively low prevalence of RCoVs in Thailand (9/435; 2.07%) based on RT‐PCR detection of the RdRp gene. The detection of RCoVs in Thailand highlighted the persistent circulation of the *Betacoronavirus muris* species, *Embecovirus* subgenus, within the *Betacoronavirus* genus in rodents. The RCoVs were detected only in three provinces, sampled across three geographic regions in Thailand, indicating low‐level circulation within rodent populations. This pattern was consistent with endemic maintenance of coronaviruses in rodent reservoirs, as previously described in studies from China and other parts of Asia [3, 25–28]. Such endemic circulation supported the role of rodents as long‐term reservoirs contributing to coronavirus diversity and evolution [29, 30].

The observed prevalence of RCoV (2.07%) was consistent with previous surveillance studies in rodents across Asia, where detection rates typically range from 1%–10%, depending on geographic location, host species, and sampling strategy. For example, in previous studies in Cambodia, Lao PDR, and Thailand, a low prevalence of RCoVs of ~0.3%–1.4% was reported [27, 29, 31]. In contrast, studies in Vietnam have reported rates as high as 23.5% [25]. Moreover, the prevalence observed in Thailand appears lower than that reported in large‐scale surveillance studies in China, where RCoV detection rates have ranged from ~4% to >15% in wild rodent populations [3, 26, 28]. Another study in China reported an RCoV prevalence of 17.8% in high‐density habitats [32]. In Europe, a study in France and Sweden reported prevalence rates of 6.4% and 3.4%, respectively, with specific regions or host populations serving as hotspots [30, 33]. These discrepancies may be attributed to variations in host species composition, environmental factors, viral circulation dynamics, and the sensitivity of molecular detection methods, which affect RCoV detection. In this study, the higher prevalence observed in the northern region compared to other regions may reflect ecological and environmental factors, including rodent density, agricultural practices, and the intensity of human–animal interactions. Similar geographic variation has been reported in studies conducted in China, where RCoV prevalence differed significantly between provinces and ecological settings [3, 28].

In this dataset, all positive swab samples were identified from *B. indica*, although host specificity requires confirmation through larger‐scale studies. Moreover, sampling bias could occur since most sampling was conducted in rice fields or nearby villages. In this study, we focused on rodent species commonly consumed by humans in Thailand. Thus, our results showed the detection of CoVs in *B. indica*, whereas CoVs were absent in *B. savilei*, *B. bengalensis*, *and R. argentiventer*. Our results were consistent with a study in Vietnam, where *B. indica* in rice fields was identified as a key host for *Embecoviruses* subgenus, including those closely related to the LRNV and the murine coronavirus [25]. This observation was consistent with several reports that members of the Muridae family serve as primary reservoirs for *Betacoronavirus muris* and other rodent‐associated coronaviruses [30]. However, the lack of statistical significance between species and RCoV prevalence in this study suggested the need for larger‐scale sampling across rodent species to confirm host specificity.

### 6.2. Genetic Relatedness of Thai RCoVs

Phylogenetic analysis in this study demonstrated that Thai RCoVs belong to the *Betacoronavirus* genus, within the *Embecovirus* subgenus, *Betacoronavirus muris species*. This classification is consistent with previously reported RCoVs identified in Asia and globally. The whole‐genome comparison indicates that Thai RCoV‐CU34984RS belongs to the rat‐associated *Embecovirus* lineage and shares a close evolutionary relationship with previously identified RtCoVs from China, Thailand, and Vietnam, suggesting the persistence and regional circulation of genetically related RtCoVs across Asia. At the gene level, pairwise analysis also revealed high nucleotide identity (up to ~97.19%) between Thai RCoVs and RCoVs from China (RtRt‐CoV‐Tk2011). Similarly, substantial genetic similarity (up to ~95.41% and 95.09%) was observed with RCoV from Vietnam (MuCoV‐VZ‐16715) and Thailand (Tl2006A), supporting the presence of a regionally distributed lineage of rodent‐associated *Embecovirus* across Southeast and East Asia [3, 25, 28, 31]. In contrast, lower nucleotide identity (~67.10%–87.03%) was observed when comparing Thai RCoVs with human‐associated *Embecoviruses* such as HCoV‐HKU1 and HCoV‐OC43. This finding indicated evolutionary divergence between rodent and human lineages, although phylogenetic studies suggested that RCoVs may represent ancestral sources for certain human *Embecoviruses* [2].

The analysis of the S revealed conserved residues in receptor‐binding regions associated with CEACAM1 and 9‐O‐acetyl sialic acid binding. These conserved motifs are characteristic of *Embecoviruses* and have been reported in RCoVs from China, suggesting functional conservation in receptor usage [3]. It has been known that 9‐O‐acetylated sialic acids are expressed in the human respiratory and enteric tracts. The optimization of the NTD for 9‐O‐acetylated sialic acid binding, characterized by the conserved W90, L80, K81, and T83, marked a significant evolutionary change in rodent‐specific CEACAM1 binding [19–21].

Amino acid variability was observed in key functional regions, including the S1/S2 cleavage site and receptor‐binding motifs. Such variation may reflect adaptive evolution to local host populations and ecological conditions, as described in recent coronavirus evolutionary studies of SARS‐CoV‐2 [34]. For the S1/S2 cleavage site, the presence of multiple basic furin cleavage sites (RRKRR or RRARR) at the S1/S2 junction (positions 806−810) warranted further investigation. These polybasic motifs are essential for S protein priming and efficient M fusion, both of which significantly enhance viral entry into human cells [23].

In this study, two RCoV strains, CU34984OS and CU34984RS, were detected in the same animal. The whole‐genome sequences of both RCoVs exhibited distinctly different genomic and evolutionary profiles. CU34984RS was identified as a natural recombinant originating from Tk2011‐like and FJ2015‐like parental lineages. On the other hand, no recombination events were detected in the whole genome of CU34984OS. This genomic divergence within the same host suggested evidence of coinfection or cocirculation of genetically distinct RCoV lineages. In the previous study, intrasubgenus (*Embecovirus*) coinfections in field rats have been documented in Vietnam [25]. In this study, the presence of recombinant RCoV (CU34984RS) and nonrecombinant RCoV (CU34984OS) in the same animal highlighted the ongoing evolutionary plasticity of RCoVs. These findings highlighted that rodents can carry complex, multilineage viral pathogens, emphasizing the need for ongoing molecular surveillance to detect new RCoV variants.

A limitation of the study was the uneven distribution of sample sizes across the surveyed regions (central: *n* = 175; northeastern: *n* = 164; northern: *n* = 70; western: *n* = 21; and southern: *n* = 5) due to convenience sampling. The small sample sizes from the western and southern regions limited the statistical power to confirm the absence of viral presence. Therefore, these results should be interpreted with caution and not as conclusive evidence of the absence of circulation in areas where the virus was not detected. Instead, our results provided preliminary insights, highlighting the need for more systematic, large‐scale, and balanced surveillance across all regions of Thailand.

In summary, this study highlighted the presence of genetically diverse RCoVs circulating at low prevalence in Thailand. The genetic data indicated that Thai RCoVs are closely related to rodent‐associated coronaviruses circulating in China and neighboring regions, supporting the hypothesis of regional viral circulation and shared evolutionary origins. The genetic variability observed in key functional regions underscores the need for continued monitoring. From a One Health perspective, integrated surveillance across wildlife, domestic animals, humans, and the environment is essential. Targeted monitoring of rodent populations in high‐risk ecological settings, combined with genomic analysis and data sharing, will enhance the early detection, risk assessment, and disease prevention and control.

## Author Contributions

Chanakarn Nasamran drafted the manuscript. Chanakarn Nasamran, Supassama Chaiyawong, Supanat Boonyapisitsopa, and Napawan Bunpapong performed the sample collection. Chanakarn Nasamran, Supassama Chaiyawong, and Kamonpan Charoenkul performed virus detection, characterization, and phylogenetic analysis and participated in the statistical analysis. Chanakarn Nasamran and Alongkorn Amonsin designed the study and revised and approved the manuscript.

## Funding

The C2F Program of Chulalongkorn University supports the first author’s (Chanakarn Nasamran) Ph.D. scholarship. This study was supported by the Thailand Science Research and Innovation Fund, Chulalongkorn University, FF69 (Grant HEA_FF69_076_3100_006). Chulalongkorn University also provided financial support to the Center of Excellence for Emerging and Re‐emerging Infectious Diseases in Animals (CUEIDAs).

## Disclosure

All authors reviewed and approved the manuscript.

## Ethics Statement

This study was conducted under the Chulalongkorn University Animal Care and Use Committee (CU‐ACUC) under Protocol Number CUIACUC# 2431085. This research followed the ARRIVE guidelines.

## Consent

The authors have nothing to report.

## Conflicts of Interest

The authors declare no conflicts of interest.

## Supporting Information

Additional supporting information can be found online in the Supporting Information section.

## Supporting information


**Supporting Information** Table S1: List of primers for whole‐genome sequencing for Thai RCoVs. Table S2: Summary of whole‐genome sequencing using Oxford Nanopore sequencing and mapping statistics for Thai RCoVs. Figure S1: Whole‐genome sequencing depth of coverage across Thai RCoVs: CU34998OS, CU36371RS, CU34984OS, CU34984RS, and CU34997RS. The horizontal red dashed line indicates the minimum depth threshold of 20× required for consensus calling. Figure S2: Phylogenetic tree of the E, M, N, and HE genes. The phylogenetic tree was constructed using the neighbor‐joining algorithm and the maximum composite likelihood model, with 1000 bootstrap replicates. Figure S3: Recombination analysis of RCoV‐CU34984RS. (A) Bootscan analysis of Thai RCoV showed the recombinant RCoV‐CU34984RS. Bootscan analysis using SimPlot v.3.5.1 (window size: 500 bp, step size: 50 bp, 1000 bootstrap replicates). (B) Phylogenetic tree of the nucleotide sequence region derived from the major parent. (C) Phylogenetic tree of the nucleotide sequence region derived from the minor parent. The black circle indicates Thai RCoV‐CU34984RS, the pink square indicates the major parent strain (RtRt‐CoV/Tk2011/China/2011), and the blue triangle indicates the minor parent strain (RtBi‐CoV/FJ2015/China/2015).

## Data Availability

The data that support the findings of this study are openly available in the GenBank database at https://www.ncbi.nlm.nih.gov/genbank/, Accession Numbers PZ438032–PZ438041.
