# Supplementary material for: Whole‐Genome Characterization of Rodent Coronaviruses (RCoVs) in Thailand, 2024–2025
Source: Transbound Emerg Dis. 2026 Aug 12;2026:7053968. doi: 10.1155/tbed/7053968 (PMC13469764; doi:10.1155/tbed/7053968)
Supplement: Supplementary file 1 [file TBED-2026-7053968-s001.docx]

**Supplement Tables**

Supplement Table 1: List of primers for whole-genome sequencing for Thai RCoVs

Supplement Table 2. Summary of whole-genome sequencing using Oxford Nanopore Sequencing and mapping statistics for Thai RCoVs.

**Supplement Figures**

Supplement Figure 1. Whole-genome sequencing depth of coverage across Thai-RCoVs: CU34998OS, CU36371RS, CU34984OS, CU34984RS, and CU34997RS. The horizontal red dashed line indicates the minimum depth threshold of 20× required for consensus calling.

Supplement Figure 2. Phylogenetic tree of the E, M, N, and HE genes. The phylogenetic tree was constructed using the Neighbor-Joining algorithm and the Maximum composite likelihood model, with 1,000 bootstrap replicates.

Supplement Figure 3. Recombination analysis of RCoV-CU34984RS. A) BootScan analysis of Thai RCoV showed the recombinant RCoV-CU34984RS. BootScan analysis using SimPlot v.3.5.1 (Window size: 500 bp, Step size: 50 bp, 1,000 bootstrap replicates. B) Phylogenetic tree of the nucleotide sequence region derived from the major parent. (C) Phylogenetic tree of the nucleotide sequence region derived from the minor parent. The black circle indicates Thai-RCoV-CU34984RS, the pink square indicates the major parent strain (RtRt-CoV/Tk2011/China/2011), and the blue triangle indicates the minor parent strain (RtBi-CoV/FJ2015/China/2015).

Supplement Table 1. List of primers for whole-genome sequencing for Thai RCoVs

| Type | Primer | Sequence (5’-3’) | Ta (℃) | Product size (bp) |
| --- | --- | --- | --- | --- |
| Outer | RCoV F_4 | AGAGTGATTGGCGTCCGTACc | 45 | 4101 |
|  | RCoV R_4104 | CTTAACAAAACACACATTTGG |  |  |
| Inner | RCoV F_4 | AGAGTGATTGGCGTCCGTACc | 45 | 1076 |
|  | RCoV R_1079 | ATCCAAATGGACTGCAACTC |  |  |
|  | RCoV F_964 | CCCATACTCTTTGTGGACCAG | 45 | 1089 |
|  | RCoV R_2052 | GCATATCCATGCACATATCAGC |  |  |
|  | RCoV F_1944 | GCTTGATGGATTGGTGCC | 45 | 984 |
|  | RCoV R_2927 | CCTTACAAGGACCAAGAGTAC |  |  |
|  | RCoV F_2820 | TCTAGATAAGGCTTGCTCTG | 45 | 1285 |
|  | RCoV R_4104 | CTTAACAAAACACACATTTGG |  |  |
| Outer | RCoV F_3908 | CAGGAAAGTCTTTATGGCTGC | 45 | 4447 |
|  | RCoV R_8354 | TTACCCTGCACATGCTTAGC |  |  |
| Inner | RCoV F_3908 | CAGGAAAGTCTTTATGGCTGC | 45 | 1206 |
|  | RCoV R_5113 | TCATACTGRTACAARACCTTACC |  |  |
|  | RCoV F_4993 | TCCAGCCTTGTTGCTGAAGG | 45 | 1277 |
|  | RCoV R_6269 | TAGGTCTATTAAAATATGTAAGCG |  |  |
|  | RCoV F_6122 | GGAGTATAGAGTGACAGAGTG | 45 | 1181 |
|  | RCoV R_7302 | TATGGTAAGCAACTGTAATCC |  |  |
|  | RCoV F_7168 | CATGAAGTTGTGAGGCATGG | 50 | 1187 |
|  | RCoV R_8354 | TTACCCTGCACATGCTTAGC |  |  |
| Outer | RCoV F_8199 | GATAACTAAGTCTATGATGTCG | 45 | 4331 |
|  | RCoV R_12529 | GCCATACATTGCCAGCATAGG |  |  |
| Inner | RCoV F_8199 | GATAACTAAGTCTATGATGTCG | 45 | 1163 |
|  | RCoV R_9364 | AATCTATAGGCCGCACTAGAC |  |  |
|  | RCoV F_9179 | CACGCTCTATGACTTATTGC | 45 | 1189 |
|  | RCoV R_10367 | ACAGTAAATGTCTCACCAGG |  |  |
|  | RCoV F_10257 | TATGTCTTACCAGATGATGG | 45 | 1231 |
|  | RCoV R_11487 | AGCAGTAGCAAGTGATAATA |  |  |
|  | RCoV F_11307 | CTATGATCTTTGTGACCATGC | 45 | 1223 |
|  | RCoV R_12529 | GCCATACATTGCCAGCATAGG |  |  |
| Outer | RCoV F_12432 | ATTAACTTCAAACACTCTGAC | 45 | 3815 |
|  | RCoV R_16246 | GCTTGCGTATGCAACTGCC |  |  |
| inner | RCoV F_12432 | ATTAACTTCAAACACTCTGAC | 45 | 1016 |
|  | RCoV R_13447 | GGGCATTTACACTTGTACC |  |  |
|  | RCoV F_13302 | CTGTGTCATATGTTCTGACGC | 45 | 1154 |
|  | RCoV R_14455 | CATCCATATTCATwACAACACC |  |  |
|  | RCoV F_14345 | TTACCTAATACCTGTTTTGGG | 45 | 1023 |
|  | RCoV R_15367 | CTTGGGCACATTCATTCGC |  |  |
|  | RCoV F_15243 | CTAAATGTGATCGTGCTATGC | 45 | 1004 |
|  | RCoV R_16246 | GCTTGCGTATGCAACTGCC |  |  |
| Outer | RCoV F_16157 | TTTAAGAAGTGCAGTGATGC | 45 | 4012 |
|  | RCoV R_20168 | CGATTACCACCTGGATTACC |  |  |
| inner | RCoV F_16157 | TTTAAGAAGTGCAGTGATGC | 45 | 1091 |
|  | RCoV R_17247 | AAACACATACTTACGAGTGG |  |  |
|  | RCoV F_17118 | CGATGCATTGTGTGAAAAGG | 45 | 1062 |
|  | RCoV R_18179 | CATCAAGGGCCAAATCAAGC |  |  |
|  | RCoV F_18011 | ATTGTAGCAAGAGTTATGTGG | 45 | 1228 |
|  | RCoV R_19238 | TTATTAACATACAAACTACCACC |  |  |
|  | RCoV F_19117 | ATGTTTTGGAACTGCAATGTGG | 45 | 1052 |
|  | RCoV R_20168 | CGATTACCACCTGGATTACC |  |  |
| Outer | RCoV F_20051 | CCAAAGTGGAATTTTGGTTTGC | 45 | 3875 |
|  | RCoV R_23925 | ACCCTTTAATGCGAGATTTCG |  |  |
| Inner | RCoV F_20051 | CCAAAGTGGAATTTTGGTTTGC | 45 | 1228 |
|  | RCoV R_21278 | CTAGAAGACGCATTTACATTGG |  |  |
|  | RCoV F_21076 | CTTACTAAGAACATTGGGGAG | 45 | 1124 |
|  | RCoV R_22199 | CAATAGCCACAAATAGCACC |  |  |
|  | RCoV F_22067 | CTAGGCTTGAGTTAGTGAAGC | 45 | 919 |
|  | RCoV R_22985 | AACCAGTGAGAGTAAAATTAGC |  |  |
|  | RCoV F_22821 | CGCTCACTATTGTAAATGTTCC | 45 | 1105 |
|  | RCoV R_23925 | ACCCTTTAATGCGAGATTTCG |  |  |
| Outer | RCoV_S_1F | GGTGTTAGGCTTCATGAAGC | 45 | 4527 |
|  | RCoV_S_8R | GACGACCAGAATTAAATATAGG |  |  |
| Inner | RCoV_S_1F | GGTGTTAGGCTTCATGAAGC | 45 | 632 |
|  | RCoV_S_1R | CAATCAACATTAACATCATAAG |  |  |
|  | RCoV_S_2F | GTGAATTTAATACTATAGTTATAGG | 45 | 779 |
|  | RCoV_S_2R | CATACCATACACTTTAGAAGC |  |  |
|  | RCoV_S_3F | TCTCCTCTCAATTGGGAACG | 45 | 718 |
|  | RCoV_S_3R | CATGAGACCAGCCTACAAAGG |  |  |
|  | RCoV_S_4F | GTGTTAATGAAGATAAGTGTGG | 45 | 711 |
|  | RCoV_S_4R | TTTGTATCTCATATAAACCACC |  |  |
|  | RCoV_S_5F | TTGGGTTGTGTTGTTAATGC | 45 | 848 |
|  | RCoV_S_5R | TCCATAGTGACACCCAAACC |  |  |
|  | RCoV_S_6F | TGTGTGCAATCTTTTAATGG | 45 | 769 |
|  | RCoV_S_6R | AATAAGAACTACCAGTGAAC |  |  |
|  | RCoV_S_7F | GGTTAATTTCTGTGGCAATGG | 45 | 654 |
|  | RCoV_S_7R | TACTCATCACAACAATTACC |  |  |
|  | RCoV_S_8F | CATCAATCTCAAGGAAATTGG | 45 | 648 |
|  | RCoV_S_8R | GACGACCAGAATTAAATATAGG |  |  |
| Outer | RCoV F_27527 | AAATTGGTACATATGAAATGTATG | 45 | 3631 |
|  | RCoV R_31157 | TGTGATTCTTCCAATTGGCC |  |  |
| Inner | RCoV F_27527 | AAATTGGTACATATGAAATGTATG | 45 | 1050 |
|  | RCoV R_28576 | TACCACACTGTGTCTGTCGG |  |  |
|  | RCoV F_28443 | GTTTCACTTGCTACGGAAGC | 50 | 1124 |
|  | RCoV R_29566 | AGCGGTTTCCAGAGGAGC |  |  |
|  | RCoV F_29331 | AACTGTGGCTAAGGTTCAGC | 50 | 1248 |
|  | RCoV R_30578 | CTTATTGCACCTGAATAGCGC |  |  |
|  | RCoV F_30422 | CTAGTGATCCACAGTTCCCC | 45 | 736 |
|  | RCoV R_31157 | TGTGATTCTTCCAATTGGCC |  |  |

Supplement Table 2. Summary of whole-genome sequencing using Oxford Nanopore Sequencing and mapping statistics for Thai RCoVs.

| ID | Reads | Mapped reads | Mean length | GC% | Mean coverage | S.D. |
| --- | --- | --- | --- | --- | --- | --- |
| CU34984RS | 772,545 | 606,212 | 30,157 | 37.53% | 18,274.48 | 30,533.06 |
| CU34984OS | 600,442 | 540,426 | 31,083 | 37.23% | 18,969.33 | 12,883.09 |
| CU34998OS | 324,022 | 268,841 | 31,083 | 37.03% | 9,266.46 | 6,212.50 |
| CU34997RS | 350,319 | 318,937 | 31,352 | 37.96% | 10,133.55 | 10,469.93 |
| CU37361RS | 720,849 | 514,149 | 30,837 | 36.44% | 14,400.02 | 26,650.30 |

Supplement Figure 1. Whole-genome sequencing depth of coverage across Thai-RCoVs: CU34998OS, CU36371RS, CU34984OS, CU34984RS, and CU34997RS. The horizontal red dashed line indicates the minimum depth threshold of 20× required for consensus calling.

Supplement Figure 2. Phylogenetic tree of the E, M, N, and HE genes. The phylogenetic tree was constructed using the Neighbor-Joining algorithm and the Maximum composite likelihood model, with 1,000 bootstrap replicates.

Supplement Figure 3. Recombination analysis of RCoV-CU34984RS. A) BootScan analysis of Thai RCoV showed the recombinant RCoV-CU34984RS. BootScan analysis using SimPlot v.3.5.1 (Window size: 500 bp, Step size: 50 bp, 1,000 bootstrap replicates. B) Phylogenetic tree of the nucleotide sequence region derived from the major parent. (C) Phylogenetic tree of the nucleotide sequence region derived from the minor parent. The black circle indicates Thai-RCoV-CU34984RS, the pink square indicates the major parent strain (RtRt-CoV/Tk2011/China/2011), and the blue triangle indicates the minor parent strain (RtBi-CoV/FJ2015/China/2015).
